# Congenital Malaria in a 2-Day-Old Neonate: A Case Report and Literature Review

**DOI:** 10.1155/2021/9960006

**Published:** 2021-07-07

**Authors:** Dickson Kajoba, Walufu Ivan Egesa, Habonimana Jean Petit, Muhiadin Omar Matan, Goretty Laker, William Mugowa Waibi, Daniel Asiimwe

**Affiliations:** ^1^Department of Paediatrics and Child Health, Faculty of Clinical Medicine and Dentistry, Kampala International University, Kampala, Uganda; ^2^Department of Surgery, Faculty of Clinical Medicine and Dentistry, Kampala International University, Kampala, Uganda

## Abstract

Congenital malaria is the presence of malaria parasites in a blood smear obtained from a neonate usually within 24 hours to 7 days of life. It has for long been regarded a rare condition. However, recent data indicate that congenital malaria complicates around 35.9% of live births globally, 0–37% in Sub-Saharan Africa and about 4–6.1% in Eastern Uganda. We present a 2-day-old neonate who presented with fever, irritability, and failure to breastfeed. Laboratory tests indicated that the neonate had a positive Giemsa-stained peripheral smear for *Plasmodium falciparum*, with a positive malaria rapid diagnostic test (MRDT) for *P*. *falciparum* malaria. The mother had a negative peripheral film for malaria and a negative MRDT. The neonate was managed with intravenous artesunate with improvement.

## 1. Introduction

Uganda remains a highly malaria-endemic country, one of the 6 most affected countries contributing 5% to the global malaria burden [1]. Children under 5 years and pregnant women are the most affected with children under 5 years contributing an estimated 70% of malaria-related deaths in the country [2].

Congenital malaria (CM) is defined as a positive blood smear for malaria in a neonate from 24 hours to 7 days of life. This is usually due to maternofoetal transfer of malaria parasites. On the other hand, neonatal malaria is defined by the presence of malaria parasites in peripheral blood within 28 days of life, usually attributable to mosquito bites [3, 4]. The maternofoetal transfer of malaria parasites can be reduced if mothers routinely take their intermittent presumptive treatment of malaria during pregnancy with sulfadoxine/pyrimethamine which reduces neonatal mortality by approximately 60% [5].

CM presents with nonspecific signs and symptoms of fever, anaemia, jaundice, vomiting, lethargy, convulsions, irritability, tachypnoea, respiratory distress, and hepatosplenomegaly which overlap with sepsis syndrome [6]. Due to a low index of suspicion and nonspecific presentations, it is often wrongly managed as neonatal sepsis, which contributes to mortality and morbidity among neonates [7].

Neonates have some level of protection from malaria due to passive immunisation from maternal antibodies, presence of foetal haemoglobin, and low iron levels which do not favour growth of plasmodium [3]. However, as maternal IgG and HbF wane, there is a surge in malaria parasitaemia among the infants [8, 9]. The prevalence of congenital malaria may be under-reported due to its nonspecificity in presentation and delay in onset of symptoms. Only 34% of affected neonates become symptomatic within 72 hours of life, while others may present after 3 weeks of life [8].

Currently, it is estimated that there is a 33.7% global burden of congenital malaria [10] with Sub-Saharan Africa having a prevalence of 0 to 37% [11] and a prevalence of 4–6.1% in Eastern Uganda [12, 13]. In order to reduce the malaria case incidence and death rate to at least 90%, the World Health Organisation (WHO) came up with Global Technical Strategy for Malaria, 2016–2030. The program launched in 2015 was geared towards eliminating malaria in at least 35 countries and to prevent its reintroduction in all countries that eliminated it [14].

## 2. Case Report

We report a case of a male neonate delivered at term by spontaneous vaginal delivery from a lower level health centre, with a birthweight of 3.7 kg. Apgar score was not documented, but the neonate cried immediately after birth. Labour lasted about 8 hours and membranes ruptured just before delivery. Breastfeeding was initiated within the 1^st^ hour of life and the neonate was suckling well. He passed meconium and urine within the first 24 hours of life.

The neonate was born to a 29-year-old G2P1 + 0 who attended antenatal care 6 times, starting at 3 months. She had been screened for HIV, syphilis, hepatitis B, and urinalysis, and all were negative, with a blood group of A Rhesus D negative. She received tetanus toxoid, Fansidar for malaria prophylaxis, haematinics, and deworming. She reported that the pregnancy was uneventful.

About 36 hours after delivery, the neonate developed a high-grade fever, irritability, poor breastfeeding, and yellowing of eyes and skin. The neonate was managed with unknown oral medication before referral to Jinja Regional Referral Hospital for further management.

At admission, the neonate was conscious, with an axillary temperature of 38.0°C, and jaundice (Kramer stage 2). There was no respiratory distress, no pallor, no dehydration, and no dysmorphic features noted. The systemic physical examination was unremarkable.

Investigations done were as follows:

1. Malaria rapid diagnostic test (MRDT) for *P*. *falciparum* was positive; thin blood smear showed *P*. *falciparum* malaria species with 1.846^*∗*^10^3^ malaria-infected red blood cells.
2. Complete blood count was normal with Hb of 15.2 g/dl, white blood cells 11.79^*∗*^103/ul, and platelet count 244^*∗*^103/ul.
3. Blood group was A Rhesus D negative.
4. Total serum bilirubin was 200.33 umol/l with direct bilirubin 7.48 umol/l.
5. The rapid diagnostic test for *P*. *falciparum* was negative, and no haemoparasites were seen on the peripheral smear of maternal blood.

The neonate was initiated on intravenous artesunate at admission, 12 hr and 24 hr later, then once a day for 5 days. Intravenous empiric antibiotics (cloxacillin and cefotaxime) for presumed neonatal sepsis were also administered. After 48 hours of antibiotics, C-reactive protein (CRP) was done and it was nonreactive, and the antibiotics were stopped. Blood culture and sensitivity was not performed for this patient. A peripheral blood smear done on day 5 of antimalarial treatment revealed no malaria parasites. The neonate was discharged after 5 days of artesunate with great improvement. We were not able to follow up the patient after discharge.

## 3. Discussion

Malaria is a parasitic disease that is transmitted by an infectious female Anopheles mosquito during a blood meal. This is caused by a parasitic protozoan of the genus plasmodium which has 5 different species, with *P*. *falciparum* being the most predominant in the Sub-Saharan region [1]. In this case report, the neonate had *P*. *falciparum* as the causative plasmodium species.

In 2019, there were 229 million cases of malaria, with over 94% of an estimated 409,000 deaths globally. More than 94% of all cases and deaths occurred in Sub-Saharan Africa. Children under 5 years are the most vulnerable, and in 2019, they accounted for 67% (274000) of all malaria deaths worldwide [15].

Congenital malaria is defined as the presence of malarial parasites in the peripheral blood smear of the new born from 24 hours to 7 days of life. However, it can occur beyond 28 days of life confounding with neonatal malaria [3, 6]. CM is acquired from the mother, while neonatal malaria is by mosquito inoculation [6]. Unfortunately, due to its nonspecific presentations and low index of suspicion, CM is often managed as neonatal sepsis, which unwittingly increases hospital stay and neonatal morbidity and mortality [7].

Babies are believed to have partial protection from malaria in the first few months of life, owing to passively acquired maternal IgG antibodies, the predominance of haemoglobin F (HbF) in their erythrocytes, and the low levels of iron and para-amino benzoic acid (both required for parasite growth) in breast milk [3]. Despite these factors, newborns and infants less than 12 months of age are one of the most vulnerable groups affected by malaria [16].

Globally, there is a 33.7% prevalence of congenital malaria with Africa having 39.5%. Furthermore, it is estimated that 40 neonates per 1000 live births will experience clinical malaria during the first 7 days of life [10]. In Sub -Saharan Africa, the prevalence is estimated to be between 0 and 37% [4], while in Eastern Uganda, the prevalence of CM is reported to range from 4 to 6.1% [12, 13].

The true burden of congenital malaria may be underestimated due to absence of routine screening of newborns with fever, low index of suspicion, absence of specific signs, and symptoms, coupled with the late symptom presentation. Only one-third (34%) of the affected neonates present within 72 hours of life [9, 13]. This was the case with our patient who presented with CM within 48 hours of life. On the other hand, delayed presentation of CM has been reported up to 2 months after delivery [3]. A case report by Rai and colleagues documented a 21-day-old neonate with congenital malaria in Burundi [17]. This is because transplacentally acquired maternal antibodies delay the onset of symptoms, with symptom occurrence coinciding with half-life of maternal IgG antibodies [17].

It is recommended therefore that malaria parasite testing be included in the routine screening of febrile babies with suspected septicaemia in malaria-endemic regions [18]. To maximise the chances of detection, some authors suggest malaria screening irrespective of clinical presentation at delivery, followed by weekly follow up with repeated blood smear up to 4 weeks if the mother was known to have malaria 7 days before delivery [9]. Neonates with severe illness and parasitaemia should have blood samples taken for culture and, in any case, should be treated with antibiotics as well as antimalarial drugs [3].

Use of peripheral blood smear with microscopy is the routinely used diagnostic modality for diagnosis of malaria in developing countries [13], but with the introduction of malaria rapid diagnostic testing (MRDT), there has been found a useful alternative with good sensitivity and specificity for malaria diagnosis including pregnant women and newborns. Polymerase chain reaction (PCR) may have a higher sensitivity for CM. However, it is not readily available in developing countries, except in research settings [19]. In this case, the MRDT and blood smear for maternal and neonatal blood were negative and positive for *P*. *falciparum,* respectively. PCR could have been useful in differentiating between congenital or neonatal malaria since it is very sensitive compared to MRDT and peripheral smear.

Malaria due to *P*. *falciparum* should be treated, be it symptomatic or asymptomatic because it is associated with high mortality and morbidity [20]. However, there are no clear guidelines for treatment of CM [9, 10]. Artemisinin-based combination therapy (ACT) is the regimen by WHO, yet there are limited clinical trials particularly in neonates for ACT, and many carry labels restricting its use [9]. WHO recommends use of same dosages for neonates below 5 kgs as the dosage for neonates weighing 5 kgs [16]. This however caries a risk of drug over dose in these neonates. Additionally, there is no well-established pharmacokinetics and pharmacodynamics of these antimalarial drugs in neonates with still rapidly evolving physiology. Therefore, parenteral treatment is preferred in neonates and young infants [9], as was the case in this neonate, where parenteral artesunate was considered.

The prognosis of congenital malaria is variable, depending on when intervention is initiated. In Burkina Faso, Nagalo and colleagues reported that 11.8% of CM-related deaths occurred within an average of 4.8 days from admission, of which 55% of these deaths occurred within 24 hours of admission [21]. However, timely intervention of the newborn may prevent neonatal morbidity and mortality [17], as was the case in this neonate.

## 4. Conclusion

Congenital malaria should be considered as a differential for sepsis in neonates presenting with unexplained fever and failure to breastfeed. There is need to raise awareness of this condition so as to increase diagnostic suspicion and investigatory habit among patients suspected for neonatal sepsis.
